# Determinants of Physical Activity among Children with Disabilities

**DOI:** 10.3390/healthcare11040494

**Published:** 2023-02-08

**Authors:** Salmah Alghamdi, Rasha Alsaigh

**Affiliations:** Maternity and Childhood Nursing Department, Faculty of Nursing, King Abdulaziz University, P.O. Box 80200, Jeddah 21589, Saudi Arabia

**Keywords:** physical activity, disabilities, social determinants, children

## Abstract

Maintaining children’s physical activity levels is crucial to preventing obesity and improving their health and well-being. However, achieving the recommended daily level of 60 min of moderate-to-vigorous intensity physical activity can be difficult for children with disabilities. Moreover, children with disabilities spend less time engaging in physical activity than their typically developing peers. This study aimed to assess the personal, environmental, and social determinants of physical activity among children with disabilities. This quantitative, cross-sectional study was conducted through an online survey of a convenient sample, including 125 parents of children with disabilities aged between 5 and 18 years from different regions in the Kingdom of Saudi Arabia. Approximately 40.8% of the participants were aged between 41 and 50 years, and 57.6% (the participants and friends of their children) did not engage in regular exercise. Statistically significant differences were observed between the perception of children’s health and physical activity summary scores and the engagement of children’s friends in physical activity and physical activity summary scores. Efforts should be made to reinforce parents’ perceptions of their children’s health regarding physical activity and to support the social determinants of physical activity that ensure their children’s friends’ engagement. Specialized interventional studies are needed to support parents with children.

## 1. Introduction

The physical, mental, and psychological health advantages of physical activity (PA) have been well documented in the literature. The World Health Organization suggests that youth should have at least 60 min a day of moderate-to-vigorous PA (MVPA) intensity [1]. However, achieving this amount of daily PA can be difficult for children with disabilities. The Ministry of Health in the Kingdom of Saudi Arabia defines disability as “a physical, sensory, mental, communicative, educational, or psychological impairment that has a substantial and long-term adverse effect on the person’s ability to perform normal day-to-day activities” [2]. However, several barriers hinder the PA of children with disabilities, including inadequate programs, time, and/or disability [3]. A review showed that obesity among children with disabilities is two times that of their non-disabled peers [4]. Moreover, it has been previously suggested that children with disabilities engage in less PA than their peers without disabilities [5]. Therefore, further studies should explore the determinants of PA among children with disabilities.

The physical, mental, and psychological health advantages of PA have been well documented. For example, PA enhances body composition, bone health, cognitive function, attention, and psychosocial health and prevents chronic diseases in children, including those with disabilities [6,7,8,9,10,11,12].

Liou et al. [13] reported that approximately 75% of people with specific disabilities do not participate in sufficient PA to gain its benefits. Studies show that children with disabilities spend more time engaging in sedentary activities than their peers without disabilities [14]. They also participate less in formal physical exercise [15,16,17]. Furthermore, this sedentary lifestyle with low PA levels has been linked to greater use of digital screen time and body mass index (BMI) [18], which can increase the risk of developing other health conditions such as obesity, diabetes, pressure injuries, and contractures [19]. In addition to lifestyle factors, parents may excessively use digital screens and junk food to reinforce good behavior in children with disabilities [20,21].

It has been noted internationally that children, including adolescents with varying disabilities, such as sensory, intellectual, and physical disabilities, spend most of their school days less active than their normal peers and are less likely to participate in their favorite activities, which does not comply with the WHO recommendation [22,23,24,25,26,27].

Several barriers were found in various studies that hinder the level of PA of children with disabilities, including inadequate time and/or type of disability, the child’s gender and age, level of PA, insufficient knowledge and skills, child preferences, fear, parental style, stigma, scant facilities and programs, high costs, and lack of transport [3,25,28,29]. Additionally, facilitators such as the child’s willingness to participate in PA, family, peer, and teacher support, caregivers’ educational background and fondness for PA, available facilities, the proximity of location, competent staff, sufficient information, disability form, and cultural views were also found [24,25,26].

Parents play a vital role when it comes to evaluating the participation of children with disabilities in PA. Notably, when they have numerous motives to encourage their children with disabilities to participate in PA, several barriers and hurdles, such as the child’s form of disability, insufficient time, and programs and stigma, limit their capacity to do so [3]. Moreover, their perception and opinions can directly affect their children with disabilities’ activity levels, as it was found that parents who believed that PA was not beneficial considered more hurdles to PA [30,31].

In the Kingdom of Saudi Arabia, studies have mainly focused on PA in children with Down syndrome (DS). Alhusaini et al. [32] explored PA levels in children with DS and compared them with those in healthy children. They found that children with DS were less involved in the recommended PA levels than children without DS and had higher BMIs. Alwahaibi and Aldugahishem [33] and Alghamdi et al. [34] explored mothers’ perspectives on PA among Saudi children with DS by exploring their PA levels, their needs, benefits of PA, facilitators, and barriers to caring for their children, emphasizing the need to enhance PA in children with DS.

Children and adolescents with disabilities are less physically active than their normally developing peers and encounter many barriers such as child age and gender, type of disability, level of PA, parents’ lack of time and knowledge, style of parenting, limited facilities and supporting programs, high cost of services, and transportation hurdles [3,25,28,29]. However, to the best of our knowledge, no study has explored the determinants of PA among children with disabilities, generally, in the Kingdom of Saudi Arabia. Therefore, this study aimed to assess the personal, environmental, and social determinants of physical activity among children with disabilities. A study identical to this one is crucial for children with disabilities in the Kingdom of Saudi Arabia to help understand the various factors that impact children with disabilities’ engagement in PA and develop strategies to improve PA levels in this particular population.

## 2. Materials and Methods

### 2.1. Study Design, and Settings

This was a quantitative, cross-sectional study. It was conducted through an online survey that included the parents of children with disabilities from different regions of the Kingdom of Saudi Arabia. The online survey was distributed via social media. Ethical approval was obtained from the Research Ethics Committee of the Faculty of Nursing at King Abdulaziz University. In addition, ethical approval was obtained from the Ministry of Education to recruit parents through the school to participate in the online survey.

### 2.2. Participants and Sample Size

The sample was convenient. Parents of children with disabilities, including any physical, sensory, or mental disabilities, aged between 5 and 18 years, who were enrolled in the school system were invited to participate in the study and recruited. Children were excluded if they had any chronic or acute medical condition or injury that restricted their PA. G*Power [35] was used to estimate the number of participants required for the study. The input values were α = 0.05, power = 0.85, medium effect size = 0.3, and the suggested minimum sample size was 77.

### 2.3. Research Instrument

An electronic self-administered questionnaire was distributed to the parents to assist their children with disabilities in completing the questionnaire. The questionnaire included five sections, as follows: (1) demographic questions, such as age, gender, income, and education; and (2) physical activity using the Physical Activity Questionnaire for Older Children (PAQ-C) [36]. The PAQ-C is a self-administered, 9-item questionnaire based on 7-day recall. It measures general MVPA levels among elementary school children aged 8–14 years [36]. Additionally, it contains a 5-point Likert scale ranging from low to high PAs (1–5). The final PAQ-C composite score is the result of the mean of the 9-item questionnaire, whereas item 10, used solely to detect students who had unusual events or health statuses that influenced their activity during the previous week, was removed from the questionnaire since it was pre-determined in the study’s inclusion criteria. Previous studies revealed that the PAQ-C scale is reliable and valid, with item scale correlations ˃ 0.30 and acceptable reliability for females (α = 0.83) and males (α = 0.80) [37]. The PAQ-C questionnaire is recommended to assess the pattern of PA among children with disabilities, including autism spectrum disorder, DS, and cerebral palsy [38]. It also assesses the PA in pediatric patients undergoing organ transplantation [39]. Therefore, to adapt to the Saudi Arabian culture, the following three activities from item 1 were removed: ice skating, cross-country skiing, and ice hockey/ringette.

The content validity of the Arabic-translated version of the PAQ-C scale was assessed by three Arabic-speaking experts in the field of pediatric nursing with doctoral degrees. The items of the PAQ-C scales were evaluated on a 10-point rating scale ranging from 1 (not relevant) to 10 (very relevant), according to the relevance of the items to the construct. The item-content validity index was 0.84, indicating good validity.

Furthermore, the following three items in the questionnaire were adopted from a previous study [40]: perceived health, environmental safety, and social influence. The perceived health was assessed by asking the question: “How do you describe your child’s health?”, the options to answer ranged on a scale of four from “poor” to “excellent”. The environmental safety was assessed by asking parents to describe how safe they feel walking alone in the neighborhood, the responses included: unsafe, safe sometimes, and unsafe. Social influence was assessed by inquiring about the friends’ engagement of the children with disabilities in regular exercise. Finally, experts in pediatric healthcare evaluated the questionnaire’s content validity.

### 2.4. Data Analysis

Data were analyzed using the IBM SPSS statistics 28 (Armonk, NY, USA) for Windows software. Descriptive statistics are presented as frequencies and percentages for categorical variables, while the median with interquartile range (IQR) was used for numerical variables (different calculated scores). The Kolmogorov–Smirnov test and histogram were used to assess the normality of the distribution of the outcome variables. The Mann–Whitney and Kruskal–Wallis tests were performed to determine the difference in scores of different items across the participants’ different characteristics. Statistical significance was set at *p* ≤ 0.05.

## 3. Results

### 3.1. Demographic Characteristics

Overall, 125 participants were included in the study, as shown in Table 1. Among the parents who completed the questionnaire, 86.4% were mothers. Most participating parents (40.8%) were aged between 41 and 50 years, and 28.8% were aged between 31 and 40 years. More than half of the children included were females (55.2%). Additionally, most of the study participants were married (87.2%). Regarding the monthly income, the highest percentage of the participants (34.4%) earned between Saudi riyal (SAR) 3001 and SAR 8000, and 24% earned ≤SAR 3000. However, most participants were unemployed (67.2%). Furthermore, most participants (40.8%) had a bachelor’s degree, and most children included in this study were in grade 6 or above (39.2%).

The type of education of most study participants was governmental (69.6%). More than half of the participants (89.6%) had siblings. Among the children included in the study, 37.6%, 24.8%, 12.8%, 10.4%, and 7.2% had intellectual disabilities (IDs), learning difficulties, sensory disabilities, DS, and physical disabilities, respectively.

### 3.2. Parent’s Perceptions Regarding Neighborhood Safety, Child Health, and Social Determinants

As shown in Table 2, 37.6% of the participants felt unsafe walking alone in their neighborhood. The participants perceived their children’s health as very good (48.8%) or excellent (25.6%). However, more than half of the participants’ children’s friends did not engage in regular exercise (57.6%).

### 3.3. Physical Activity Questionnaire

Table 3 shows the average score of item 1 in the PA questionnaire. The median of item 1 was 1.28 (IQR = 0.44). The median of the composite score average of items 2–8 and that of item 9 was 2 (IQR = 1.14) and 2 (IQR = 1.57), respectively. The median of the PAQ-C summary score was 1.89 (IQR = 0.85).

### 3.4. Comparison of Physical Activity Questionnaire Summary Score and Other Variables

The Mann–Whitney and Kruskal–Wallis tests were performed to determine the presence of differences in the PA questionnaire summary score across the personal characteristic categories of the participants. As shown in Table 4, a statistically significant difference was found in the number of households categories; a higher score of PA was found with the number of households < 5 (median = 2.20, IQR = 1.02) than with those with a higher number of households including 5–10 persons (median = 1.79, IQR = 0.83) (*p* = 0.004). However, as presented in Table 4, there was no statistically significant difference in the PA questionnaire summary score across other demographic variables.

Although no statistically significant differences were observed in the PA questionnaire summary scores across the perception of neighborhood safety, a statistically significant difference was observed across the categories of the perception of a child’s health, as shown in Table 5. Parents who perceived their child’s health as excellent (median = 2.09, IQR = 0.97) or very good (median = 1.97, IQR = 0.81) had higher scores than those who perceived it as good (median = 1.54, IQR = 0.97) (*p* = 0.005).

Furthermore, a statistically significant difference was observed regarding whether the children’s friends engaged in PA (*p* = 0.001). The participants whose children’s friends engaged in PA had a higher median score (median = 2.16, IQR = 0.83) than those whose children had none of their friends who engaged in PA (median = 1.65, IQR = 0.81).

## 4. Discussion

PA is associated with various health benefits in children with or without disabilities. This study has initiated an assessment of the determinants of PA among children with disabilities in Saudi Arabia, which has not been widely assessed.

This study’s results showed a statistically significant difference in physical activity questionnaire summary scores (PAQ-C) among the categories of the number of households. Specifically, a higher PAQ-C was observed with <5 households than was observed with a higher number of households, including 5–10 persons. The reason could be that parents in larger households have limited time and support to assist their children with disabilities in participating in PA. It was reported that insufficient time is a barrier that may hinder PA in children with disabilities [3]. This could also be an indicator of inadequate facilities and support available for families caring for children with disabilities in the country, particularly those who have additional responsibilities and may require additional support, which is a notable finding in many previous studies such as [3,25,26].

A statistically significant difference was observed between the perception of a child’s health and PAQ-C. Parents who perceived their child’s health as excellent or very good scored higher than those who perceived it as good. This could be because parents relate their child’s health to their willingness to participate in PA. The healthier the child, the more active they are. Consistent findings were noted in a previous study by Shields et al. [25]. Additionally, parents perceive their child’s health subjectively, which may be strongly correlated with cultural views previously determined as a factor affecting PA involvement in adolescents with disabilities in Kenya [26]. Furthermore, parents of a child with a disability that may delay their motor skills are less healthy, which could be linked to a study by Lakes et al. [30], who noted that parents who believed the PA was not beneficial experienced more difficulties in the PA and were more likely to have children with delayed motor skills. Moreover, parents’ perception of competence in their child’s physical ability has been identified as a key factor in promoting PA behaviors in children with disabilities [41].

Furthermore, a significant difference was found between whether the children’s friends engaged in PA or PAQ-C in this study. This finding is consistent with a study by Bukhala and Mogaka [26], who explored the determinants of PA participation in adolescents with disabilities in Kenya and found that peer support was a facilitator. This finding was also reported in a systematic review by Shields et al. [25].

Additionally, although no significant difference was observed between the perception of neighborhood safety and PAQ-C, most parents (37.6%) in the study, who had more mothers (86.4%), felt unsafe walking alone in the neighborhood. Accordingly, fear was perceived as a barrier in a systematic review by Shields et al. [25].

The children’s and parents’ gender, age, educational level, parents’ marital and employment status, household income, child’s disability form, having siblings, and residency location showed no significant differences with PAQ-C in this study.

Having said that, low parental level of education, low social economic status, and single-parent family were all previously associated with reduced participation levels. However, the literature has shown that environmental and family support might be more significant factors of the participation in the physical activity [42,43]. The number of siblings was not previously associated with the level of participation, which is consistent with the findings of this study [44].

Although type or form of disability showed no significant relation to the level of participation in this study, a resent metanalysis showed a disparity in the participation level according to the type of disability with it being less in those with multiple impairments [45].

Therefore, environmental and family factors and support may be more significant determinants of participation than the children’s and parents’ characteristics [42,46]. However, the lack of a significant association with PAQ-C in this study might have been due to the small sample size. In addition, providing governmental social aid to Saudi Arabian children with disabilities may explain the non-significant differences with the demographic variables in the current study as it may limit the variations among the participants.

This study’s findings should be disseminated to inform stakeholders to provide resources to support the parents of children with disabilities. The availability of facilities, recreational centers, and specialized programs may also provide convenient participation in safe PA and practical suggestions to healthcare professionals, schools, and parents to promote participation [47]. Safety combined with convenient recreational areas in each neighborhood may also promote PA. Additionally, providing family support to families with bigger households, such as respite care recreational programs, counseling, and social support coordination, could reduce the responsibility load on parents [43], which may help increase their willingness to assist in engaging their children with disabilities in PA.

This study had some limitations. Although the generalizability of the findings is possible, there were no causal inferences to be made because this was a cross-sectional study. Lastly, the PA questionnaire may have been susceptible to recall bias.

## 5. Conclusions

This study aimed to assess the personal, environmental, and social determinants of PA among children with disabilities. The findings revealed that a higher score for PA was observed with a smaller number of households (less than 5). Therefore, this study’s results highlight the need for tailored intervention studies to support parents with many children. Additionally, statistically significant differences were found regarding PAQ-C across categories of the parent’s perception of a child’s health and the engagement of their children’s friends in PA. Therefore, efforts should also be made to reinforce parents’ perceptions of their children’s health regarding PA, given that their perception is found to make a significant difference. Moreover, it is recommended to support the social determinants of PA that ensure the engagement of children with disabilities and their friends in different types of PA. This can be arranged through school programs, community campaigns, and coordinated access to public physical activity places.

## Figures and Tables

**Table 1:** Personal characteristics of the participants.

Characteristics	Frequency	Percentage
**Child’s age, year**		
Between 5 and 8	30	24
Between 9 and 11	43	34.4
Between 15 and 18	52	41.6
**Number of households**		
<5 persons	31	24.8
From 5 to 10 persons	89	71.2
˃10 persons	5	4
**Child’s educational level**		
Kindergarten	13	10.4
Grade 1	19	15.2
Grade 2	7	5.6
Grade 3	15	12
Grade 4	8	6.4
Grade 5	14	11.2
Grade 6 or above	49	39.2
**Type of education**		
Governmental	87	69.6
Private	37	29.6
**Having a sibling**		
No	13	10.4
Yes	112	89.6
**Type of disability**		
Intellectual	47	37.6
Learning difficulties	31	24.8
Sensory	16	12.8
Down syndrome	13	10.4
Physical	9	7.2
Others	9	7.2

**Table 2:** Parent’s perception regarding neighborhood safety, child health, and social determinant.

	N	%
Perception regarding the neighborhood safety		
Unsafe	47	37.6
Safe	37	29.6
Safe sometimes	41	32.8
Perception of a child’s health		
Poor	2	1.6
Good	30	24
Very good	61	48.8
Excellent	32	25.6
Friends’ engagement in regular exercise		
None	72	57.6
Some of them	53	42.4

**Table 3:** Physical activity questionnaire scores (PAQ-C).

Items	Mean	SD	Median	IQR
Composite score of Item 1 (physical activity in the spare time)	1.42	0.47	1.28	0.44
Composite score of items 2–8	2.02	0.78	2.00	1.14
Composite score of item 9	2.26	0.97	2.00	1.57
PAQ-C activity summary score	1.90	0.62	1.89	0.85

**Table 4:** Comparison of physical activity questionnaire summary score across demographics.

PAQ-C Activity Summary Score					
	N	Median	IQR	Test Statistic U/H	*p*-Value
**Parent**					
Mother	108	1.90	0.88	849.5	0.622
Father	17	1.87	0.91		
**Sex of Child**					
Male	56	1.87	1.00	1913.5	0.927
Female	69	1.92	0.84		
**Type of education**					
Governmental	87	1.80	0.90	1325.5	0.121
Private	37	0.45	1.02		
**Having a sibling**					
No	13	2.13	0.89	669.5	0.636
Yes	112	1.87	0.87		
**Parent’s age, year**					
Between 20 and 30	7	1.92	1.25	4.24	0.236
Between 31 and 40	36	2.08	0.91		
Between 41 and 50	51	1.79	0.71		
51 and above	31	1.95	1.17		
**Child’s age, year**					
Between 5 and 8	30	1.99	0.94	4.45	0.108
Between 9 and 11	43	1.95	0.72		
Between 15 and 18	52	1.65	0.99		
**Parent’s marital status**					
Married	109	1.91	0.87	0.39	0.822
Divorced	11	1.85	0.86		
Widowed	5	1.61	0.97		
**Parent’s employment status**					
Student	4	2.46	0.28	3.89	0.274
Not working	84	1.90	0.97		
Working part-time	6	1.85	0.26		
Working full-time (at least 35 h per week)	31	1.87	0.75		
**Location**					
East regions	5	1.98	0.84	1.44	0.836
Middle regions	12	2.04	1.05		
North regions	5	1.56	0.82		
South regions	12	1.75	0.80		
Western regions	91	1.89	0.97		
**Numbers of households**					
<5 persons	31	2.20	1.02	11.0	0.004
From 5 to 10 persons	89	1.79	0.83		
˃10 persons	5	0.95	1.74		
**Household’s monthly income**					
≤SAR 3000	30	1.84	0.95	0.48	0.976
SAR 3001–SR8000	43	1.92	0.88		
SAR 8001–SR 13000	20	1.84	0.66		
˃SR13000	25	1.87	0.98		
I do not know	7	1.73	0.85		
**Parent’s educational level**					
Below high school	40	1.65	0.87	2.80	0.424
High school	30	1.82	0.78		
Bachelor’s degree	51	1.98	1.04		
Master’s degree or higher	4	2.04	1.20		
**Child’s educational level**					
Grade 1	19	2.32	0.69	8.06	0.234
Grade 2	7	1.84	1.20		
Grade 3	15	1.79	0.61		
Grade 4	8	2.01	0.87		
Grade 5	14	1.75	0.72		
Grade 6 or above	49	1.71	0.97		
Kindergarten	13	1.92	0.61		
**Type of disability**					
Intellectual	47	1.92	0.68	7.0	0.220
Learning difficulties	31	1.97	0.89		
Sensory	16	1.50	1.14		
Down syndrome	13	1.89	1.26		
Physical	9	1.52	1.27		
Others	9	2.16	0.92		

**Table 5:** Comparison of physical activity questionnaire summary score across categories of parent’s perception.

PAQ-C Activity Summary Score					
	N	Median	IQR	Test Statistic U/H	*p*-Value
**Perceptions regarding neighborhood safety**					
Unsafe	47	1.79	0.87	2.36	0.307
Safe	37	1.88	1.03		
Safe sometimes	41	1.93	0.81		
**Perception about child’s health**					
Poor	2	1.28		12.8	0.005
Good	30	1.54	0.79		
Very good	61	1.97	0.81		
Excellent	32	2.09	0.97		
**Friends’ engagement in regular exercise**					
None	72	1.65	0.81	1215.5	0.001
Some of them	53	2.16	0.83		

## Data Availability

The datasets will be available on reasonable request.
